# Effects of Transcutaneous Electrical Nerve Stimulation with Taping on Wrist Spasticity, Strength, and Upper Extremity Function in Patients with Stroke: A Randomized Control Trial

**DOI:** 10.3390/jcm13082229

**Published:** 2024-04-12

**Authors:** Kyoung-sim Jung, Jin-hwa Jung, Hwi-young Cho, Tae-sung In

**Affiliations:** 1 Department of Physical Therapy, Gimcheon University, Gimcheon 39528, Republic of Korea; 20240049@gimcheon.ac.kr; 2 Department of Occupational Therapy, Semyung University, Jecheon 27136, Republic of Korea; otsalt@semyung.ac.kr; 3 Department of Physical Therapy, College of Health Science, Gachon University, Incheon 21936, Republic of Korea

**Keywords:** stroke, spasticity, tens, taping, upper extremity

## Abstract

**Objective:** Six months after the onset of stroke, over 60% of patients experience upper limb dysfunction, with spasticity being a major contributor alongside muscle weakness. This study investigated the effect of transcutaneous electrical nerve stimulation (TENS) with taping on wrist spasticity, strength, and upper extremity function in patients with stroke. **Methods:** In total, 40 patients with stroke were included and randomly divided into two groups: the TENS + taping (*n* = 20, age 52.4 ± 9.3 (range: 39 to 70)) and TENS (*n* = 20, age 53.5 ± 10.8 (range: 39 to 74)) groups. All subjects performed 30 sessions of task-related training, which included 10 min of postural control training and 20 min of task performance. Additionally, all subjects received TENS on the spastic muscle belly for 30 min before task-related training. In the TENS + taping group, taping was additionally applied to the forearm and wrist but not in the TENS group. The Modified Ashworth Scale was used to measure spasticity, and a handheld dynamometer was used to measure muscle strength. The Fugl–Meyer Assessment of Upper Extremity was used to evaluate the functional ability of the upper extremity. **Results:** In the TENS + taping group, spasticity and upper extremity function were significantly improved as compared to those in the TENS group (*p* < 0.05). However, no significant difference in muscle strength was observed between the two groups (*p* > 0.05). **Conclusions:** This study demonstrated that the combination of TENS and taping for spasticity and function of the upper extremity was more effective in relieving the spasticity than TENS alone. Therefore, we suggest this combination as an additional treatment for spasticity and function of the upper extremity.

## 1. Introduction

The hand and wrist play an essential role in upper extremity function, enabling complex movements through coordination between the muscles and the central nervous system [1]. Proper hand and wrist movements require adequate strength of the muscles involved in the movement and the timing of their activation. Spasticity, a movement disorder caused by central nervous system damage, leads to intermittent or sustained involuntary muscle activation due to disinhibition of muscle reflexes [2]. Spasticity is assessed based on the range of resistance exhibited during passive movement, and it is a common complication that interferes with the coordination, contraction of antagonist and agonist muscles, and muscle strength recovery in patients [3]. In patients with stroke, upper limb spasticity makes it challenging to attain a full range of motion of the shoulders, elbows, wrists, and finger flexors, which can interfere with daily functions, such as reaching, grasping, and releasing [4]. Immediately after a stroke-related injury, plastic changes such as reorganization occur in the central nervous system. Still, unfortunately, intact recovery of upper limb function is minimal in most stroke patients. The efficiency of the networks in the cerebral hemispheres refers to how efficiently information exchange is performed, and it is one of the important metrics of brain function [5]. A study investigating the functional networks of the damaged cerebral hemisphere in stroke patients reported active reorganization during the first 3 months post-onset, which was associated with improved motor function. However, no significant changes in neural reorganization were reported in both cerebral hemispheres after 3 months [6]. Six months after such an injury, approximately 60% of the patients reported functional impairment of the upper extremities [7].

The treatment for stroke-induced spasticity can involve physical therapy, medications, or surgery [8]; however, these options often have side effects such as sedation, confusion, nausea, muscle weakness, and liver toxicity [9]. Non-invasive treatments, such as splinting, taping, casting, or muscle stretching, can also be effective; however, the frequency and duration of their use are debatable [10]. Transcutaneous electrical nerve stimulation (TENS) is a non-invasive method for pain management with relatively few side effects [11]. Spasticity arises from the disinhibition of the dorsal reticulo-spinal tract, leading to hyper excitability of the stretch reflex mediated by Ia fibers [12]. TENS inhibits synaptic transmission by stimulating large-diameter afferent nerve fibers, thereby reducing co-contraction of spastic antagonists [13,14]. Also, continuous excitation of peripheral sensory nerve fibers can decrease corticomotor neuron excitability and render them insensitive to central excitation, which would reduce spasticity [15,16]. In a meta-analysis study on TENS, it was suggested that to enhance the spasticity-relieving effects, it is necessary to apply higher frequency (90–100 Hz), longer session duration (>30 min), and longer intervention duration (>2 weeks) [17]. However, the application time for electrotherapy, including TENS, is limited for the purpose of spasticity relief. Because the expression of titin and collagen proteins is different in spastic muscles [18], maintaining a shortened posture in the muscles can have a significant effect on microstructural changes, even in a short time [19,20].

In a meta-analysis study on upper limb function in stroke patients, it was reported that the kinesiology tape was effective in pain relief and improving range of motion [21]. However, there were differences between the studies analyzed in the literature regarding the body part and application method of kinesiology tape, the condition of the subjects, and the duration of the intervention. This raises the need to investigate the effectiveness of different kinesiology taping methods for interventions such as pain, muscle strength, joint range of motion, and upper extremity function in stroke patients and to compare these effects. Also, most studies aiming for spasticity relief utilized the functional taping technique, where the kinesiology tape was applied to antagonistic muscles to stretch the spastic muscles [22,23,24]. Recently, several studies addressed the use of additional relieving treatments for spasticity, such as stretching, casting, and taping, following Botox injections [10,23,24]. One study suggested that taping may be more effective than stretching in relieving spasticity [10], because taping can enhance the internalization of botulinum toxin type A and positively affect the viscoelastic properties of spastic muscles [24]. TENS application on the peroneal nerve of patients with stroke and plantar flexor spasticity for 30 min, followed by taping, has been shown to be more effective in relieving spasticity than TENS alone [25]. However, most studies on the combined effects of spasticity treatments have been conducted on the lower limbs [10]. A meta-analysis on the application of taping to the upper extremities demonstrated that it was effective for shoulder pain, subluxation, disability, upper extremity function, and range of motion [26]; however, the evidence for its effects on spasticity was reported to be weak due to a lack of related studies. We hypothesized that the simultaneous application of TENS and taping in stroke patients would be effective in improving spasticity, strength, and motor function compared to TENS alone. We felt it was necessary to determine these effects, particularly at the wrist, which is the most limiting limitation to activities of daily living in stroke patients. 

Therefore, this study aimed to determine the effects of TENS and taping on upper extremity spasticity, muscle strength, and upper extremity function in patients with stroke.

## 2. Experimental Section

### 2.1. Participants

**Figure 1:**
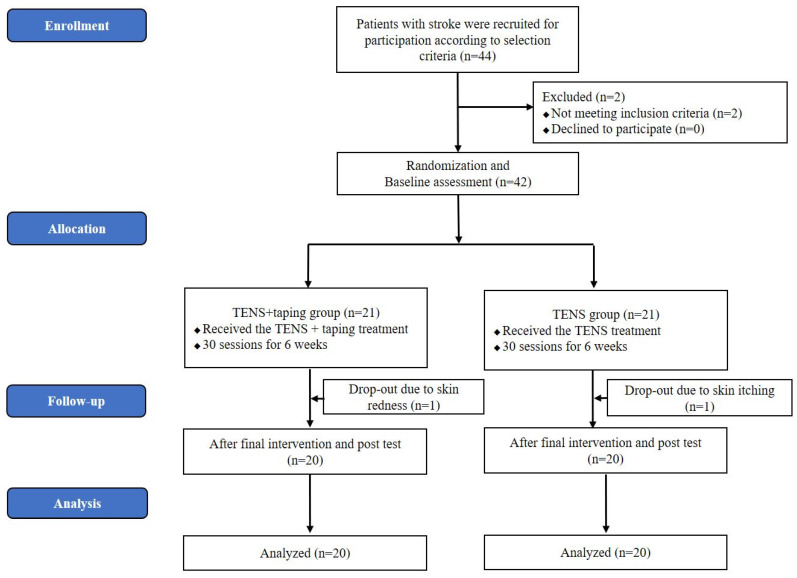
CONSORT flow diagram.

This single-blind, randomized clinical trial recruited 42 inpatients with hemiparetic stroke from a local rehabilitation center. The inclusion criteria for the participants were stroke-related hemiparesis, moderate motor impairment in the upper extremity and hand (Brunnstrom stage 3), mild to moderate spasticity (Modified Ashworth Scale (MAS) ≥ 1^+^), onset of <12 months, intact visual and vestibular function, and ability to communicate. Patients with pre-existing neurological disorders, progressive diseases, traumatic brain injury, cardiopulmonary complications, or severe sensory deficits were excluded from the study. Prior to data collection, the patients signed a consent form explaining the experimental protocol. All procedures were conducted according to the regulations of the Institutional Review Board of Gachon University (1044396-202104-HR-074-01) and registered with the Clinical Research Information Service in compliance with World Health Organization regulations (KCT0006262). In this study, G-power 3.1.7, with an effect size of 0.8 and an α error of 0.05, was used to calculate the sample size. According to this analysis, a minimum sample size of 20 participants was required for each group. In this trial, 44 participants were recruited, considering the dropout rate. Forty-four patients with chronic stroke volunteered to participate in this study. Two patients could not follow the study protocol because they did not meet the inclusion criteria. All patients were randomly assigned to (1) the TENS + taping group (*n* = 21) and (2) the TENS group (*n* = 21). Random allocation was performed using Microsoft Excel for Windows by a researcher blinded to participant recruitment. While TENS and taping are generally known to have minimal side effects, occasional adverse reactions such as burns, itching, pain, abnormal sensations, and other dermatitis have been reported. Two participants dropped out because of skin itching and redness, and forty participants underwent evaluation after training (Figure 1). All data were measured by the same physical therapist before and after the 6-week intervention period. No significant difference was found in general characteristics between the TENS + Taping and TENS groups before treatment (Table 1). The average age of the TENS + taping group was 52.4 ± 9.3 (range: 39 to 70), while the average age of the TENS group was 53.5 ± 10.8 (range: 39 to 74).

### 2.2. Intervention

All patients underwent task-related training (TRT), which consisted of 30 sessions over 6 weeks. TRT is a therapeutic program that involves functional training, ranging from simple tasks to advanced movement patterns, which include posture adjustment, shoulder mobility, and upper limb weight-bearing exercises for 10 min. Proper alignment of the upper extremity was facilitated by performing various functional tasks for 20 min, including lifting objects from a table, pouring water from a kettle into a cup, and turning off a faucet using wrist and finger movements. The size, shape, and weight of the objects varied, and as the functional performance improved, the number of repetitions increased to promote activity within each task. The therapist assisted the participants according to their abilities. 

**Figure 2:**
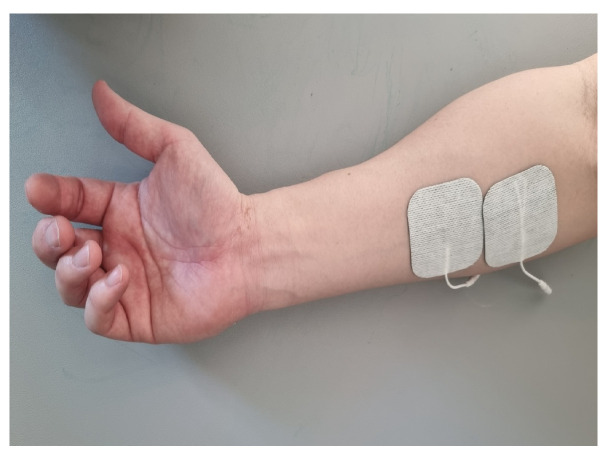
Attachment of TENS electrodes. TENS was applied to the muscle belly of the wrist flexor-induced spasticity in stroke patients for 30 min at an intensity of sensory threshold.

Both groups received TENS (TENS-7000, Koalaty Products Inc., Seattle, WA, USA) at twice the sensory threshold for 30 min prior to TRT treatment. TENS electrodes (5 cm^2^) were attached to the muscle belly of the wrist flexor (Figure 2), and a pulse phase of 50 μs and frequency of 100 Hz were applied [25]. In general, visible muscle contraction occurs when electrical stimulation is applied at an intensity twice the sensory threshold. To ensure the safety of the participants, the training was supervised by a physical therapist with at least 5 years of clinical experience. 

**Figure 3:**
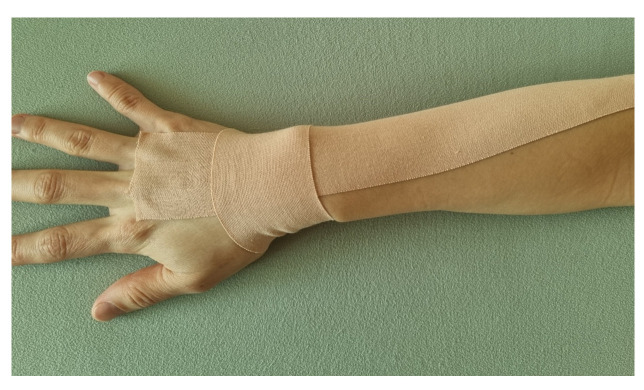
Taping attachment. Kinesiology tape, an elastic tape, was attached to the wrist extensor of a stroke patient by stretching it about 50–75% from its original length.

In the TENS + taping group, a 5 cm wide elastic tape (3NS Kinesiology tape, TS Inc., Korea) was stretched to 50–75% of its original length and attached from the origin to the insertion of the wrist extensor muscle during the training period, with the wrist maximally extended [24] (Figure 3). An elastic tape of approximately 15 cm in length was wound around the wrist in one round, starting from the radial side of the wrist. This tape was replaced once daily. The training was discontinued if severe redness, blisters, or discomfort occurred. 

### 2.3. Outcome Measure

We measured spasticity of the wrist flexors using the MAS, which is an established and reliable instrument that uses a 6-point ordinal scale to score the average resistance to passive movements for each joint [27]. Isometric muscle strength was quantified using a handheld dynamometer (DMD, JT-AA104, JTech, Chester Springs, PA, USA). To measure the wrist extensor muscle, the participants were instructed to sit comfortably and place their forearms on a table. While the participant isometrically contracted the wrist extensors, the examiner fixed the forearm and placed a handheld dynamometer on the dorsal side of the metacarpophalangeal joint. With a 1 min interval for rest between each test, three tests were conducted. The Fugl–Meyer Assessment of Upper Extremity (FMA-UE), consisting of 33 items, was used to evaluate the recovery of upper limb movements. The upper limb section includes reflex, movement observation, grasping, and coordination assessments. The scale ranges from 0 to 66, with higher scores indicating higher levels of function. The inter-rater reliability (r = 0.98–0.99) and test–retest reliability (r = 0.99) of the FMA-UE have been estimated [28].

### 2.4. Data Analysis

Statistical analyses were performed using SPSS (version 23). Normal distribution of the data was tested using the Shapiro test. The independent t-test (for continuous variables) and Chi-squared test (for categorical variables) were used to compare the baseline characteristics between the experimental and control groups. The differences before and after the intervention were compared using the paired-sample *t*-test, and the differences between the two groups were compared using the independent *t*-test. Results were considered statistically significant at *p* < 0.05.

## 3. Results

### 3.1. Changes of Spasticity

After training, spasticity in the TENS + taping group (change values, −0.6 ± 0.4 score) showed significantly greater improvement as compared to that of the TENS group (change values, −0.2 ± 0.4 score) (*p =* 0.004) (Table 2). 

### 3.2. Changes in Muscle Strength

After training, wrist strength significantly improved only in the TENS + taping group (*p* < 0.001), and no significant difference was observed between the groups (*p* > 0.05) (Table 3).

### 3.3. Changes in Upper Extremity Function

The upper extremity function was significantly improved in the TENS + taping group (change values, 8.0 ± 5.2 score), as compared to the TENS group (change values, 2.7 ± 1.2 score) (*p* < 0.001) (Table 4).

## 4. Discussion

This study investigated the effects of combined application of TENS and taping on the wrist flexor of stroke patients on spasticity, muscle strength, and upper extremity compared to TENS alone and found the following results. First, combining the two interventions reduced wrist spasticity more effectively. Second, TENS alone and the combined application of TENS and taping improved isometric muscle strength in the wrist of stroke patients, but there was no significant difference between the two methods. Third, the combined application of the two interventions improved the function of the upper extremity of stroke patients more significantly.

First, our results for spasticity showed that both methods reduced spasticity in the wrist of stroke patients, with the combined application of TENS and taping having a more significant effect than TENS alone. TENS is known to alleviate spasticity through various mechanisms such as reciprocal inhibition, reducing stretch reflex excitability, and increasing presynaptic inhibition [29]. According to the guidelines for patient management after BTX-A injection, active management following the injection can decrease the time needed to alleviate spasticity, with techniques like splinting, casting, taping, stretching, and electrical stimulation proving effective [10]. A meta-analysis examining the spasticity-relieving effect of TENS discovered that applying it to the muscle belly of spastic muscles for over 30 min effectively alleviated spasticity [17]. Additionally, a study comparing the spasticity-relieving effects of TENS, ultrasound, and paraffin reported that all three treatments had similar efficacy in relieving spasticity [30]. In this previous study, TENS was administered to the muscle belly of the plantar flexor for 30 min in both groups, resulting in significant improvements in stiffness for both. Hence, it is suggested that TENS could serve as an alternative to other modalities like ultrasound, paraffin, and stretching for alleviating spasticity. However, the protein properties of spastic muscles can change their microstructure within a short time, if the muscle maintains its shortened position [19,20]. Therefore, in this study, taping was applied to compensate for the short application time of TENS. A meta-analysis of TENS for relief from spasticity reported that combining TENS with other treatments could further enhance its effectiveness [11]. Additionally, TENS combined with taping was found to reduce plantar flexor spasticity to a significantly greater extent than only TENS [25]. Because taping continuously stretches the muscle, it causes self-inhibition of the hypertonic muscle [4]. Carda et al. reported that adding taping to botulinum toxin type A treatment strengthened the spasticity relief effect due to an increase in application time that allowed the muscles to remain stretched for a longer duration [24]. Numerous studies have investigated the efficacy of TENS or taping in relieving spasticity in the lower extremities, and we found the same effect in the upper extremities. Additionally, we found that combining TENS and taping is more effective in relieving spasticity, possibly due to the protein properties of the muscle.

In this study, changes in muscle strength after training were also measured. In the TENS + taping group, a significant change in muscle strength before and after training was observed; however, no significant differences were noted between the groups. Previous studies have reported that taping can improve muscle strength by activating cutaneous receptors or promoting muscle activity through improved blood flow. However, the results of studies on the effectiveness of taping for improving muscle strength are controversial [31]. We speculated that the difference in muscle strength between the groups after training was insignificant in our study because the taping could have been ineffective in improving the upper limb muscles. Unlike lower extremity movements that require exertion of a significant force, such as weight-bearing, upper extremity training for task-specific movements requires precise and delicate muscle control. Therefore, taping may not synergistically improve muscle strength. Another reason for the lack of significant improvement in muscle strength in our study may be the low sensitivity of the handheld dynamometer used. Unlike EMG, which can measure muscle activation, handheld dynamometers can be challenging to use for evaluating small changes in muscle strength. Therefore, even if the muscle strength improved through parallel training with TENS and taping, the change could not be accurately detected using the handheld dynamometer because the range of improvement was not large.

A meta-analysis of the effects of taping on spasticity in stroke patients reported positive effects of taping on shoulder pain, upper extremity stiffness, and range of motion [21]. Unfortunately, previous studies have not yet demonstrated the effect of taping on upper extremity function in stroke patients. Therefore, this study combined taping and TENS on the upper extremity, the main area where stroke patients have difficulty performing activities of daily living, to determine if the synergistic effect of the two treatments could improve upper extremity function. The study showed that combining both interventions significantly improved upper limb function in stroke patients compared to taping alone (Table 4). In spastic muscles, coordination and control of upper limb movements are disrupted or interrupted due to the simultaneous contraction of agonist and antagonist [4]. The improvement in upper extremity function observed in the TENS + taping group compared to the control group in this study is likely due to the effectiveness of TENS in reducing spasticity through mechanisms such as presynaptic inhibition to improve coordination. In addition, Kim et al. reported that taping can modulate muscle tone in the applied muscles to balance agonists and antagonists during voluntary movement [32]. Another reason for improving upper limb movement may be due to improved proprioception. Huang et al. reported that taping effectively relieves upper limb stiffness and improves function, which is speculated to be due to the stimulation of the sensory–motor and proprioceptive systems on the skin at the application site, resulting in improved function [4]. In the literature, the Minimum Clinically Important Difference (MCID) of the Fugl–Meyer Assessment for Upper Extremity (FMA-UE) in chronic stroke patients has been reported to be 5.3 points, reflecting the fact that much of the upper extremity function recovery occurs during the acute phase [33]. However, studies focusing on subacute stroke patients with severe functional impairment have estimated the MCID to be higher, ranging from 9 to 10 points [34]. The participants in this study were in the subacute stage and exhibited mild to moderate functional impairment before undergoing training. While there was a statistically significant difference in the change in FMA-UE scores before and after training, the increase in FMA-UE score in the TENS + taping group was only 8.0 points. This relatively small difference may be attributed to the mildness of upper limb dysfunction in our study population. To accurately assess the effect of TENS combined with taping on upper limb function, further investigation with subjects exhibiting more severe dysfunction would be necessary.

We hypothesized that the combined application of TENS and taping in stroke patients would more effectively reduce the upper extremity spasticity and improve hand motor function compared to TENS alone. This study demonstrated that the combined intervention of TENS and taping effectively reduced spasticity and improved motor function in the upper extremity compared to TENS alone. We compared the effect of the two interventions on muscle strength and found that both interventions improved the muscle strength of the wrist, but there was no significant difference between the groups. However, as we included only patients with subacute disease with onset <12 months, generalization of the results is difficult. Therefore, repeating these experiments in patients with chronic stroke is necessary in the future. Additionally, any improvement in the quality of movement after relief from spasticity, through motion analysis, needs to be studied.

## 5. Conclusions

This study confirmed the effectiveness of TENS and taping in relieving upper extremity spasticity. Furthermore, we found that the combined application of both treatments was more effective in relieving spasticity than either treatment alone. These results suggest that TENS and taping work synergistically to promote muscle coordination and balance, leading to improved relief from spasticity. Overall, this study provides evidence for the potential benefits of combining TENS and taping as a treatment approach for managing upper extremity spasticity.

## Figures and Tables

**Table 1:** Common and clinical characteristics of the subjects (N = 40).

Variables	TENS + Taping Group (*n* = 20)	TENS Group (*n* = 20)	*p*
Sex (male/female) Type (infarction/hemorrhage)	13/7	14/6	0.736 ^b^
	12/8	11/9	0.749
Involved side (left/right)	9/11	10/10	0.752
Age (years)	52.4 ± 9.3 ^a^ (39–70)	53.5 ± 10.8 (39–74)	0.755 ^c^
Height (cm)	165.4 ± 9.6	166.0 ± 10.4	0.851
Weight (kg)	62.6 ± 8.1	63.8 ± 8.3	0.634
Stroke duration (months)	8.1 ± 2.1	8.2 ± 2.4	0.889
MAS	2.1 ± 0.4	2.1 ± 0.5	0.575

PPTT, Posterior pelvic tilt. MAS, Modified Ashworth scale. ^a^ mean ± standard deviation, ^b^ chi-square test, ^c^ independent *t*-test.

**Table 2:** Changes in spasticity of the study participants (N = 40).

	TENS + Taping Group		Difference	TENS Group		Difference	*p*-Value
	Pretest	Posttest		Pretest	Posttest		
MAS (score)	2.1 ± 0.4	1.5 ± 0.5 *	-0.6 ± 0.4	2.1 ± 0.5	1.8 ± 0.6 *	−0.2 ± 0.4	0.004

TENS, Transcutaneous electrical nerve stimulation; MAS, modified Ashworth scale. * Significant difference between pretest and posttest (*p* < 0.05).

**Table 3:** Changes in muscle strength of the study participants (N = 40).

	TENS + Taping Group		Difference	TENS Group		Difference	*p*-Value
	Pretest	Posttest		Pretest	Posttest		
Muscle strength (kg)	4.4 ± 3.0	5.9 ± 4.7 *	1.5 ± 3.0	5.5 ± 4.2	6.7 ± 4.4	1.2 ± 2.9	0.737

TENS, Transcutaneous electrical nerve stimulation. * Significant difference between pretest and posttest (*p* < 0.05).

**Table 4:** Changes in FMA-UE of the study participants (N = 40).

		TENS + Taping Group		Difference	TENS Group		Difference	*p*-Value
		Pretest	Posttest		Pretest	Posttest		
FMA-UE (score)	Shoulder/Elbow/Forearm	26.5 ± 4.1	31.3 ± 3.0	4.8 ± 2.1	27.3 ± 3.5	29.3 ± 5.5	2.0 ± 2.0	
	Wrist	3.4 ± 2.8	5.3 ± 3.0	1.9 ± 3.5	4.1 ± 2.5	4.2 ± 3.3	0.1 ± 1.4	
	Hand	4.2 ± 2.4	5.0 ± 2.1	0.8 ± 2.5	4.4 ± 3.1	4.9 ± 2.4	0.5 ± 2.1	
	Coordination	3.1 ± 1.2	3.6 ± 1.1	0.5 ± 0.5	3.3 ± 1.2	3.4 ± 2.1	0.1 ± 1.5	
	Total	37.2 ± 5.5	45.2 ± 4.1 *	8.0 ± 5.2	39.1 ± 6.8	41.8 ± 6.9 *	2.7 ± 1.2	<0.001

TENS, Transcutaneous electrical nerve stimulation; FMA-UE, Fugl–Meyer Assessment of Upper Extremity. * Significant difference between pretest and posttest (*p* < 0.05).

## Data Availability

The data presented in this study are available on request from the corresponding author.
